# Chromatin Remodeler TaSWI3D Controls Wheat Susceptibility to Pathogenic Fungus *Blumeria graminis forma specialis tritici*

**DOI:** 10.3390/microorganisms13122779

**Published:** 2025-12-06

**Authors:** Yixian Fu, Wanzhen Chen, Mengdi Zhang, Xiaoyu Wang, Cheng Chang

**Affiliations:** College of Life Sciences, Qingdao University, Qingdao 266071, China

**Keywords:** wheat, chromatin remodeler, powdery mildew, SA biosynthesis, epigenetic regulation

## Abstract

Pathogenic fungus *Blumeria graminis* *forma specialis* *tritici* (*B.g. tritici*) is the causal agent of the devastating wheat powdery mildew disease. Identifying the key regulators governing wheat susceptibility to the *B.g. tritici* pathogen is essential for developing wheat varieties with improved powdery mildew resistance. In this study, we demonstrated that the wheat chromatin remodeler TaSWI3D positively regulates wheat susceptibility to *B.g. tritici*. Overexpression of *TaSWI3D* gene attenuates wheat resistance against *B.g. tritici*, while silencing of *TaSWI3D* gene potentiates wheat powdery mildew resistance. TaSWI3D protein was found to be enriched at the promoter regions of the *TaSARD1* gene encoding the salicylic acid (SA) biosynthesis activator, and silencing of *TaSWI3D* resulted in decreased nucleosome occupancy at the *TaSARD1* promoter regions. Activated *TaSARD1* transcription and increased SA accumulation were observed in the *TaSWI3D*-silenced wheat plants. Silencing of *TaSARD1* and the SA biosynthesis gene *TaICS1* resulted in attenuated SA biosynthesis and decreased powdery mildew resistance in the *TaSWI3D*-silenced wheat plants. These findings support that the chromatin remodeler TaSWI3D maintains epigenetic suppression of the SA biosynthesis activator gene *TaSARD1* and negatively regulates SA biosynthesis, thereby positively contributing to wheat powdery mildew susceptibility.

## 1. Introduction

Cereal crop bread wheat (*Triticum aestivum* L.) provides nearly one fifth of the dietary calories consumed by humans. Global population growth drives the demand for wheat grains [1,2]. However, the fungal pathogen *Blumeria graminis* *forma specialis* *tritici* (*B.g. tritici*) causes wheat powdery mildew disease, leading to wheat yield losses of 5–50% [3,4,5]. In addition, *B.g. tritici* infection could affect wheat seed metabolism and development, causing reductions in the quality of grain and flour [3,4,5]. The infection of aerial pathogen *B.g. tritici* initiates with conidial germination on the epidermal surface of wheat [3,4,5]. After penetration into wheat epidermal cells, *B.g. tritici* develops the haustorium within host cells and finally forms the microcolony [3,4,5]. Breeding wheat varieties with improved resistance against *B.g. tritici* is one of the most effective and economic ways to control wheat powdery mildew disease [4,5]. To this end, it is vital to identify the key regulators that govern wheat susceptibility to the *B.g. tritici* pathogen.

By utilizing the energy of ATP hydrolysis, chromatin remodelers could alter chromatin configuration and regulate gene expression, DNA replication, and even genome stability [6,7,8]. In the crop plant rice and model plant *Arabidopsis thaliana*, several chromatin remodelers have been demonstrated to regulate the expression of defense-related genes and affect plant–microbe interactions. For instance, CHROMATIN REMODELING 11 (OsCHR11) controls nucleosome occupancy in defense-related genes and negatively regulates rice resistance to bacterial blight [9]. Arabidopsis switch/sucrose nonfermentable (SWI/SNF) chromatin-remodeling protein BAF60/SWP73A inhibits the intracellular immune receptor nucleotide-binding domain and leucine-rich repeat (NLRs) genes to attenuate plant defense [10]. Characterizing the function of chromatin remodelers in the regulation of compatible wheat–*B.g. tritici* interactions could facilitate the identification of key regulators of wheat powdery mildew susceptibility.

In this study, we characterized the wheat chromatin remodeler TaSWI3D as a positive regulator of compatible wheat–*B.g. tritici* interactions. Overexpression of *TaSWI3D* gene dampens wheat powdery mildew resistance, while silencing of *TaSWI3D* gene potentiates wheat powdery mildew resistance. TaSWI3D protein was found to be enriched at the promoter regions of the *TaSARD1* gene encoding the salicylic acid (SA) biosynthesis activator, and silencing of *TaSWI3D* resulted in decreased nucleosome occupancy at the *TaSARD1* promoter regions. Activated *TaSARD1* transcription and increased SA accumulation were observed in the *TaSWI3D*-silenced wheat plants. Silencing of *TaSARD1* and SA biosynthesis gene *TaICS1* resulted in attenuated SA biosynthesis and decreased powdery mildew resistance in the *TaSWI3D*-silenced wheat plants. These findings support that the chromatin remodeler TaSWI3D maintains the epigenetic suppression of the SA biosynthesis activator gene *TaSARD1* and negatively regulates SA biosynthesis, thereby positively contributing to compatible wheat–*B.g. tritici* interactions. This study revealed novel epigenetic regulators governing wheat SA biosynthesis and identified a new *Susceptibility* (*S*) gene for wheat resistance breeding against *B.g. tritici* pathogen.

## 2. Materials and Methods

### 2.1. Plant and Fungal Materials

*B.g. tritici*-susceptible wheat cultivar Yannong 999, employed in this study, was developed by Shandong Yantai Academy of Agricultural Sciences and was derived from a previous study [11]. The pedigree of cultivar Yannong 999 is Yanhangxuan 2/Lin 9511//Yan BLU14-15, and its released number is Shandong (2011), South region of Huang-Huai (2016), Shanxi (2018). Wheat seedlings were grown in growth chambers under a 16 h light 20 °C/8 h dark 18 °C cycle. The virulent *B.g. tritici* genotype isolate E09, derived from a previous study, remained on the wheat cultivar Yannong 999 seedlings [11].

### 2.2. Analysis of Gene Transcript Level and Transcription Rate

A reverse transcription–quantitative polymerase chain reaction (RT-qPCR) assay was conducted as previously described to analyze the gene transcript levels [11]. Expression of *TaSWI3D* was analyzed using primers 5′AAGCGCAAGGCGTCGGGGTC3′/5′GTTTCCTCG GCGGGCGTCT3′, whereas the primers for amplifying *TaEF1*, *TaSARD1*, *TaICS1*, *TaPR1*, and *TaPR2* were derived from a previous study [11]. A nuclear run-on assay was performed to analyze the gene transcription rate. For the nuclear run-on assay, wheat cell nuclei were isolated and mixed with a reaction buffer (25 mM biotin-16-UTP and 0.75 mM of ATP, CTP, and GTP) for the transcription reaction. After RNA extraction, the nascent RNA was enriched using streptavidin magnetic beads and subjected to the RT-qPCR assay.

### 2.3. Barley Stripe Mosaic Virus-Induced Gene Silencing (BSMV-VIGS) Assay

For the BSMV-VIGS assay, fragments of the *TaSWI3D* gene were amplified using primers 5′AAGGAAGTTTAATAGTTAAGGAATCATTATGC3′/5′AACCACCACCACCGTAGGAAGTACTACATGTGCAAC3′ and cloned into the pCa-γbLIC vector to create the construct BSMV-*TaSWI3D*, as described by Yuan et al. [12]. The BSMV-*TaSARD1* and BSMV-*TaICS1* constructs were derived from previous studies [11]. The BSMV-VIGS assay was conducted as previously described [12].

### 2.4. Wheat Epidermal Cell Gene Overexpression Assay

For the single-cell transient gene overexpression assay, coding regions of *TaSWI3D-4A*, *TaSWI3D-4B*, and *TaSWI3D-4D* were amplified using primers 5′GGGGACAAGTTTGTACAAAAAAGCAGGCTTCATGGAGCCCAAGTCCCAGC3′/5′GGGGACCACTTTGTACAAGAAAGCTGGGTCTCAGCTGCTCGGCCGGGGCAT3′, 5′GGGGACAAGTTTGTACAAAAAAGCAGGCTTCATGGAGCCCAAGCCCCAGC3′/5′GGGGACCACTTTGTACAAGAAAGCTGGGTCTCAGCTGCTCGGCCGGGGCA3′, and 5′GGGGACAAGTTTGTACAAAAAAGCAGGCTTCATGGAGCCCAAGCCCCAGC3′/5′GGGGACCACTTTGTACAAGAAAGCTGGGTCTCAGCTGCTCGGCCGGGGCA3′, and PCR products were cloned into pIPKb001 to create the pIPKb001-*TaSWI3D-4A* (for OE-*TaSWI3D-4A*), pIPKb001-*TaSWI3D-4B* (for OE-*TaSWI3D-4B*), and pIPKb001-*TaSWI3D-4B* (for OE-*TaSWI3D-4B*) constructs. The single-cell transient gene overexpression assay was conducted as previously described [11].

### 2.5. Analysis of Wheat–B.g. tritici Interactions

*B.g. tritici* haustorium and microcolony formation were statistically analyzed to define wheat–*B.g. tritici* interactions, as previously described [11]. The *B.g. tritici* haustorium index (HI %) was calculated as a percentage of GUS-stained wheat epidermal cells containing *B.g. tritici* haustoria, whereas the *B.g. tritici* microcolony index (MI %) was defined as a percentage of germinated *B.g. tritici* conidia containing microcolony formations.

### 2.6. Analysis of SA Amount

The SA accumulation was analyzed by High-Performance Liquid Chromatography (HPLC), as previously described [11,13]. Ortho-anisic acid was employed as the internal standard, and the free SA amount was calculated in ng per mg fresh weight (FW), with reference to the ortho-anisic acid amount.

### 2.7. Chromatin Immunoprecipitation (ChIP) and Nucleosome Occupancy Micrococcal Nuclease (MNase) Assay

The ChIP assay analyzing the enrichment of the TaSWI3D-Myc protein at *TaSARD1* gene promoter regions was conducted as previously described [11]. Briefly, the antibody α-Myc (Santa Cruz Biotechnology, sc-789) was employed for the immunoprecipitation. The nucleosome occupancy micrococcal nuclease (MNase) assay, analyzing the chromatin structure at *TaSARD1* promoter regions, was conducted as previously described [13]. The primer sequences used for the qPCR analysis of the *TaSARD1* promoter were derived from previous studies [11].

### 2.8. Phylogenetic Tree Reconstruction

The SWI3D proteins identified from *Arabidopsis thaliana*, *Brassica rapa*, *Solanum lycopersicum*, *Brachypodium distachyon*, *Zea mays*, *Oryza sativa*, and *Triticum aestivum* were aligned by Clustal W and used for the phylogenetic tree reconstruction via the Neighbor-Joining method with 2000 bootstraps.

### 2.9. Statistical Analysis

For the statistical analysis of the wheat gene transcript level, the transcription rate, the SA amount, protein enrichment, and nucleosome occupancy at the gene promoter region, as well as for the *B.g. tritici* HI % and MI %, at least three independent experiments were performed for each assay. Three technical replicates per assay were analyzed using Student’s *t*-test, and the value represents the mean ± standard error.

## 3. Results

### 3.1. Isolation of Wheat TaSWI3D Genes Based on Homology with Arabidopsis AtSWI3D

**Figure 1:**
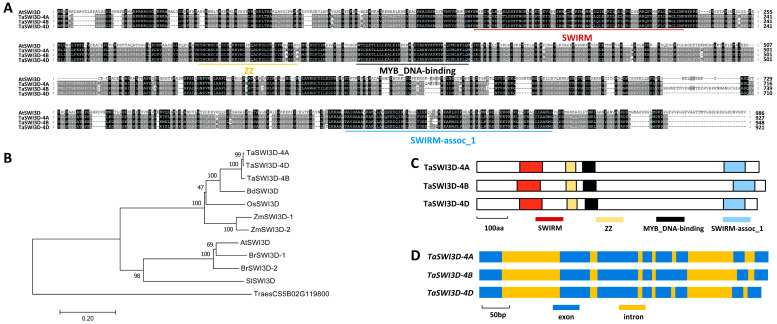
Homology-based identification of wheat TaSWI3D. (**A**) Protein sequence alignments of Arabidopsis AtSWI3D, wheat TaSWI3D-4A, TaSWI3D-4B, and TaSWI3D-4D. (**B**) Phylogenetic relationships of the SWI3D proteins to Arabidopsis (At), mustard (Br), tomato (Sl), *Brachypodium* (Bd), maize (Zm), rice (Os), and wheat (Ta). (**C**) Domain arrangement of wheat TaSWI3D-4A, TaSWI3D-4B, and TaSWI3D-4D proteins. (**D**) Genomic sequence structure of wheat *TaSWI3D-4A*, *TaSWI3D-4B*, and *TaSWI3D-4D* genes.

Herein, we aimed to explore the impact of the putative regulation of the SWI3D subunit of the wheat SWI/SNF chromatin-remodeling complex on compatible wheat–*B.g. tritici* interactions. To this end, we first employed the amino acid sequence of Arabidopsis AtSWI3D (At4g34430) as a query to search the reference genome of the hexaploid bread wheat (*Triticum aestivum* L., AABBDD). *TaSWI3D-4A* (*TraesCS4A02G261600*), *TaSWI3D-4B* (*TraesCS4B02G053000*), and *TaSWI3D-4D* (*TraesCS4D02G053300*), separately located on wheat chromosomes 4A, 4B, and 4D, were identified as wheat homologs of AtSWI3D. As shown in Figure 1A, these predicted TaSWI3D-4A, TaSWI3D-4B, and TaSWI3D-4D proteins shared more than 38% identity with Arabidopsis AtSWI3D. As shown in Figure 1B, phylogenetic analysis validated that wheat TaSWI3D-4A, TaSWI3D-4B, and TaSWI3D-4D proteins are close homologs of Arabidopsis AtSWI3D, mustard BrSWI3D, tomato SlSWI3D, *Brachypodium* BdSWI3D, maize ZmSWI3D, and rice OsSWI3D (Figure 1B). SWIRM, ZZ (Zinc finger, ZZ type), MYB_DNA-binding, and SWIRM-assoc_1 domains were identified from all TaSWI3D proteins. The coding regions of the *TaSWI3D-4A* and *TaSWI3D-4D* genomic sequences contained eight exons and seven introns, whereas the *TaSWI3D-4B* genomic sequences contained seven exons and six introns (Figure 1D).

### 3.2. Functional Analysis of TaSWI3D Genes in the Regulation of Compatible Wheat–B.g. tritici Interactions

**Figure 2:**
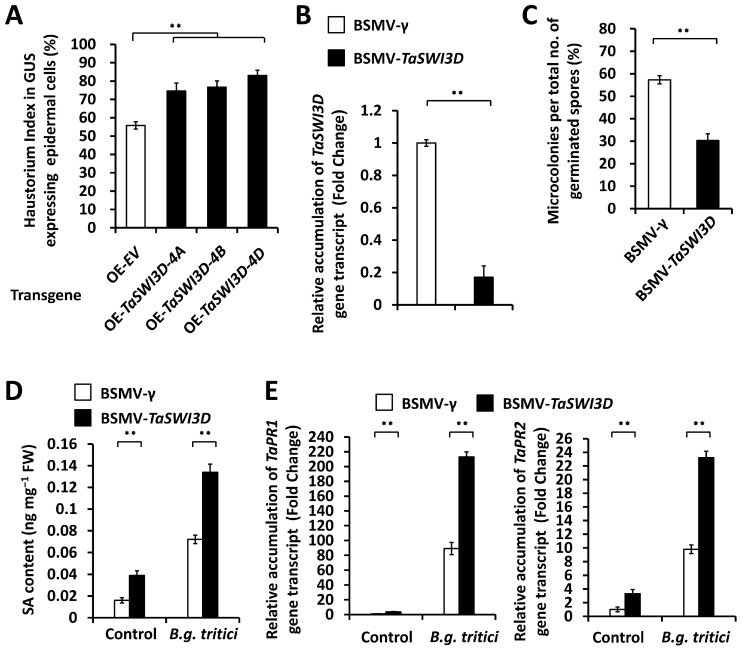
Functional characterization of the *TaSWI3D* gene in the regulation of compatible wheat–*B.g. tritici* interactions. (**A**) Analysis of *B.g. tritici* haustorium index in wheat epidermal cells overexpressing *TaSWI3D*. (**B**) RT-qPCR analysis of *TaSWI3D* gene expression levels in the wheat leaves silencing *TaSWI3D*. (**C**) Analysis of *B.g. tritici* microcolony index on wheat leaves silencing *TaSWI3D*. (**D**) Measurement of SA accumulation in the wheat leaves silencing *TaSWI3D*. (**E**) RT-qPCR analysis of *TaPR1* and *TaPR2* gene expression levels in the wheat leaves silencing *TaSWI3D*. For (**A**–**E**), three technical replicates per treatment were statistically analyzed, and data are presented as the mean ± SE (Student’s *t*-test; ** *p* < 0.01); these assays were repeated in three independent biological replicates with similar results.

To determine the impact of the potential regulation of *TaSWI3D* genes on compatible wheat–*B.g. tritici* interactions, we first transiently overexpress these *TaSWI3D-4A*, *TaSWI3D-4B*, and *TaSWI3D-4D* genes in the wheat leaf epidermal cells. *B.g. tritici* conidia were inoculated on these bombarded wheat leaves, and the formation of *B.g. tritici* haustoria was statistically analyzed. The *B.g. tritici* haustorium index (HI %) in wheat cells overexpressing *TaSWI3D-4A*, *TaSWI3D-4B*, or *TaSWI3D-4D* genes was above 74.5%, while this was about 55.6% in wheat cells transfected with the empty vector (OE-EV) control (Figure 2A). Thereafter, we silenced all endogenous *TaSWI3D* genes in the wheat leaves by employing the BSMV-VIGS technique. As shown in Figure 2B, the expression levels of the *TaSWI3D* gene were significantly reduced in *TaSWI3D*-silenced wheat leaves (Figure 2B). After inoculation with *B.g. tritici* conidia, the powdery mildew microcolony formation was statistically analyzed. As shown in Figure 2C, *TaSWI3D*-silenced wheat leaves displayed 30.2% *B.g. tritici* microcolony index (MI %) values, compared with 57.3% *B.g. tritici* microcolony index values for the BSMV-γ control wheat leaves. These data implicate that the *TaSWI3D* genes positively contribute to wheat susceptibility to the *B.g. tritici* pathogen.

The accumulating evidence supports that phytohormone salicylic acid (SA) plays important roles in wheat powdery mildew resistance [11,13,14,15]. To analyze the potential impact of the regulation of *TaSWI3D* genes on SA accumulation, we performed High-Performance Liquid Chromatography (HPLC) to analyze the SA accumulation. As shown in Figure 2D, *TaSWI3D*-silenced wheat leaves displayed significantly increased SA accumulation levels compared with BSMV-γ control wheat leaves, suggesting that TaSWI3D negatively regulates SA accumulation in bread wheat. Consistent with this, the expression levels of SA signaling marker genes *TaPR1* and *TaPR2* were significantly increased in the *TaSWI3D*-silenced wheat leaves compared with the BSMV-γ control wheat leaves (Figure 2B). These results suggest that the chromatin remodeler TaSWI3D negatively regulates SA biosynthesis and positively regulates wheat powdery mildew susceptibility.

### 3.3. Regulation of SA Biosynthesis Activator Gene TaSARD1 by TaSWI3D

**Figure 3:**
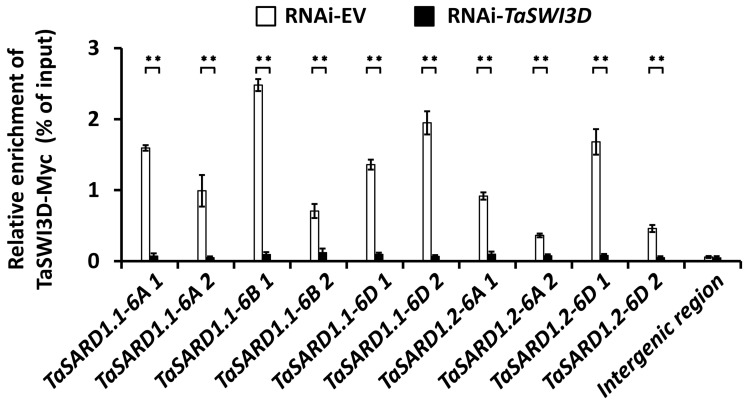
Analysis of TaSWI3D enrichment at *TaSARD1* promoter regions. ChIP-qPCR analysis of TaSWI3D-Myc enrichment at *TaSARD1* promoter regions in wheat cells. Three technical replicates per treatment were statistically analyzed, and data are presented as the mean ± SE (Student’s *t*-test; ** *p* < 0.01); these assays were repeated in three independent biological replicates with similar results.

The SA biosynthesis activator gene TaSARD1 was demonstrated to play a key role in wheat powdery mildew resistance, and *TaSARD1* gene transcription is tightly regulated at the chromatin level [11,13]. We aimed to determine whether the chromatin remodeler TaSWI3D could be enriched at the *TaSARD1* promoter regions. To examine this hypothesis, we transfected the wheat protoplast with *TaSWI3D-Myc* constructs and performed a ChIP assay to characterize the distribution of TaSWI3D-Myc at the *TaSARD1* promoter regions (Figure 3). *TaSARD1* promoter regions, previously demonstrated to be regulated by epigenetic events like histone acetylation and chromatin assembly, were chosen for the ChIP analysis. As shown in Figure 3, these *TaSARD1* promoter regions were found to be enriched in DNA samples immuno-precipitated with TaSWI3D-Myc proteins, indicating that the chromatin remodeler TaSWI3D was enriched at the promoter regions of the *TaSARD1* genes.

**Figure 4:**
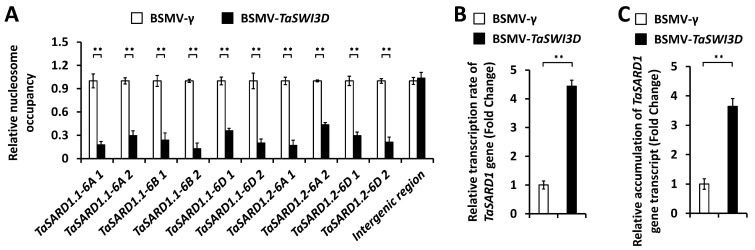
Characterization of nucleosomal occupancy and gene transcription at *TaSARD1* loci in *TaSWI3D*-silenced wheat leaves. (**A**) MNase analysis of nucleosome occupancy at *TaSARD1* promoter regions in the wheat leaves silencing *TaSWI3D*. The nucleosome occupancy levels in wheat leaves infected with the BSMV-γ empty vector (negative control) were set to 1.0. Transcription rates (**B**) and expression levels (**C**) of *TaSARD1* gene in the wheat leaves silencing *TaSWI3D* were measured by nuclear run-on and qRT-PCR assays, respectively. For (**A**–**C**), three technical replicates per treatment were statistically analyzed, and data are presented as the mean ± SE (Student’s *t*-test; ** *p* < 0.01); these assays were repeated in three independent biological replicates with similar results.

To examine whether TaSWI3D affects the chromatin structure at the *TaSARD1* promoter regions, we analyzed the nucleosome occupancy at the promoter regions of the *TaSARD1* by employing the nucleosome occupancy micrococcal nuclease (MNase) assay. As shown in Figure 4A, silencing of the *TaSWI3D* gene reduced nucleosome occupancy at the *TaSARD1* promoter regions. Nuclear run-on and qRT-PCR assays demonstrated that the transcription rate and transcript accumulation of the *TaSARD1* gene were significantly enhanced in the *TaSWI3D*-silenced wheat leaves compared with that of the BSMV-γ control wheat leaves (Figure 4B,C). These results suggest that the chromatin remodeler TaSWI3D negatively regulates *TaSARD1* gene transcription, probably via maintaining a repressive chromatin state at the *TaSARD1* gene.

### 3.4. Functional Analysis of TaSARD1 and TaICS1 Genes in TaSWI3D-Governed Wheat–B.g. tritici Interactions

**Figure 5:**
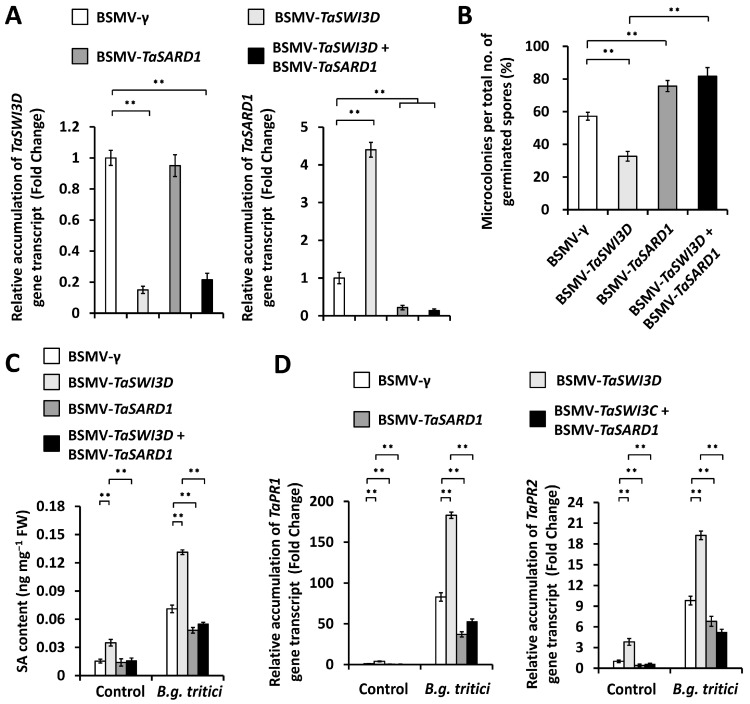
Characterization of the genetic interplay of *TaSWI3D* and *TaSARD1* in the regulation of compatible wheat–*B.g. tritici* interactions. (**A**) qRT-PCR analysis of *TaSWI3D* and *TaSARD1* expression levels in the wheat leaves silencing *TaSWI3D* and *TaSARD1* or co-silencing *TaSWI3D* and *TaSARD1*. (**B**) Analysis of *B.g. tritici* microcolony index on wheat leaves silencing *TaSWI3D* and *TaSARD1* or co-silencing *TaSWI3D* and *TaSARD1*. (**C**) Measurement of SA accumulation in the wheat leaves silencing *TaSWI3D* and *TaSARD1* or co-silencing *TaSWI3D* and *TaSARD1*. (**D**) RT-qPCR analysis of *TaPR1* and *TaPR2* gene expression levels in the wheat leaves silencing *TaSWI3D* and *TaSARD1* or co-silencing *TaSWI3D* and *TaSARD1*. For (**A**–**D**), three technical replicates per treatment were statistically analyzed, and data are presented as the mean ± SE (Student’s *t*-test; ** *p* < 0.01); these assays were repeated in three independent biological replicates with similar results.

We aimed to determine whether the characterized *TaSARD1* genes contribute to wheat powdery mildew resistance suppressed by the chromatin remodeler TaSWI3D. To examine this hypothesis, we conducted the BSMV-VIGS assay to simultaneously silence *TaSWI3D* and *TaSARD1* genes in the wheat leaves and statistically analyzed the formation of the *B.g. tritici* microcolony. As shown in Figure 5A, *TaSWI3D* and *TaSARD1*-cosilenced wheat leaves displayed decreased expression levels of the *TaSWI3D* and *TaSARD1* genes compared with the BSMV-*γ* control wheat leaves. Notably, *TaSWI3D* and *TaSARD1*-cosilenced wheat leaves showed 75.6% *B.g. tritici* microcolony index values, compared with 57.2% *B.g. tritici* microcolony index values for the BSMV-γ control wheat leaves (Figure 5B). Consistent with this, *TaSWI3D* and *TaSARD1*-cosilenced wheat leaves displayed remarkably decreased SA accumulation compared with the BSMV-*γ* control wheat leaves (Figure 5C). The RT-qPCR assay confirmed that the simultaneous silencing of *TaSWI3D* and *TaSARD1* genes resulted in a significant reduction in the expression levels of *TaPR1* and *TaPR2* genes compared with the BSMV-*γ* control (Figure 5D). These data suggest that the chromatin remodeler TaSWI3D maintains epigenetic suppression of the *TaSARD1* gene and negatively regulates SA biosynthesis, thus contributing to wheat susceptibility to *B.g. tritici*.

**Figure 6:**
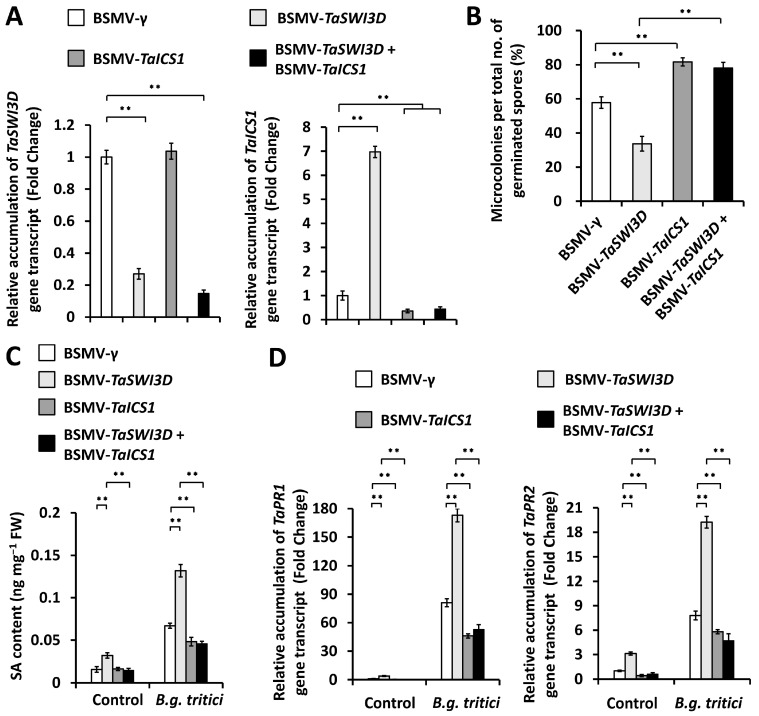
Characterization of the genetic interplay of *TaSWI3D* and *TaICS1* in the regulation of compatible wheat–*B.g. tritici* interactions. (**A**) qRT-PCR analysis of *TaSWI3D* and *TaICS1* expression levels in the wheat leaves silencing *TaSWI3D* and *TaICS1* or co-silencing *TaSWI3D* and *TaICS1*. (**B**) Analysis of *B.g. tritici* microcolony index on wheat leaves silencing *TaSWI3D* and *TaICS1* or co-silencing *TaSWI3D* and *TaICS1*. (**C**) Measurement of SA accumulation in the wheat leaves silencing *TaSWI3D* and *TaICS1* or co-silencing *TaSWI3D* and *TaICS1*. (**D**) RT-qPCR analysis of *TaPR1* and *TaPR2* gene expression levels in the wheat leaves silencing *TaSWI3D* and *TaICS1* or co-silencing *TaSWI3D* and *TaICS1*. For (**A**–**D**), three technical replicates per treatment were statistically analyzed, and data are presented as the mean ± SE (Student’s *t*-test; ** *p* < 0.01); these assays were repeated in three independent biological replicates with similar results.

We ask whether the TaICS1 (isochorismate synthase 1), a core component of wheat SA biosynthetic machinery, contributes to wheat powdery mildew resistance suppressed by the chromatin remodeler TaSWI3D [14,15]. To examine this hypothesis, we conducted a BSMV-VIGS assay to simultaneously silence *TaSWI3D* and *TaICS1* genes in the wheat leaves and statistically analyzed the formation of the *B.g. tritici* microcolony. As shown in Figure 6A, the simultaneous silencing of *TaSWI3D* and *TaICS1* resulted in a significant increase in the *B.g. tritici* microcolony index compared with BSMV-γ controls (Figure 6B). Consistently, remarkably decreased SA accumulation was observed in the *TaSWI3D* and *TaICS1*-cosilenced wheat leaves (Figure 6C). The RT-qPCR assay confirmed that the simultaneous silencing of *TaSWI3D* and *TaICS1* genes resulted in a significant reduction in the expression levels of *TaPR1* and *TaPR2* genes compared with the BSMV-*γ* control (Figure 6D). These results collectively confirm that the chromatin remodeler TaSWI3D negatively regulated powdery mildew resistance by suppressing the SA biosynthesis activated by the *TaSARD1* gene, thereby facilitating wheat susceptibility to *B.g. tritici*.

## 4. Discussion

### 4.1. The Chromatin Remodeler TaSWI3D Suppresses SA Biosynthesis and Facilitates Compatible Wheat–B.g. tritici Interactions

Herein, *TaSWI3D-4A*, *TaSWI3D-4B*, and *TaSWI3D-4B,* separately located on wheat chromosomes 4A, 4B, and 4B, were identified as wheat homologs of the Arabidopsis chromatin remodeler AtSWI3D. Overexpression of *TaSWI3D-4A*, *TaSWI3D-4B*, and *TaSWI3D-4B* resulted in increased *B.g. tritici* haustorium index values, whereas silencing of *TaSWI3D* caused decreased *B.g. tritici* microcolony index values, indicating that the chromatin remodeler TaSWI3D positively regulates wheat susceptibility to *B.g. tritici* and facilitates *B.g. tritici* haustoria development and microcolony formation. Various epigenetic regulators have been identified to regulate compatible wheat–*B.g. tritici* interactions. For instance, histone deacetylase TaHDA6 positively contributes to wheat susceptibility to *B.g. tritici*, while wheat histone acetyltransferase TaHAG1 positively regulates wheat resistance to *B.g. tritici* [16,17]. Chromatin assembly factor-1 (CAF-1) finetunes wheat susceptibility to *B.g. tritici* by suppressing SA and wax biosynthesis [11].

Previous studies have revealed that plant hormone SA plays a key role in wheat powdery mildew resistance [11,13,14,15]. In this study, we demonstrated that the chromatin remodeler TaSWI3D was enriched at the promoter regions of *TaSARD1*, an activator of SA biosynthesis, and suppresses *TaSARD1* gene transcription via maintaining a repressive chromatin state at the *TaSARD1* gene. SA overaccumulation and defense-marker gene activation were observed in the *TaSWI3D*-silenced wheat plants. Consistent with this, silencing of *TaSARD1* and SA biosynthesis gene *TaICS1* could attenuate SA biosynthesis and dampen powdery mildew resistance potentiated by the knockdown of *TaSWI3D* expression. These results suggest that the chromatin remodeler TaSWI3D negatively regulates SA biosynthesis mediated by the TaSARD1-TaICS1 module, thus suppressing powdery mildew resistance and facilitating compatible wheat–*B.g. tritici* interactions. Wheat chromatin assembly factor CAF-1 was previously identified as a negative regulator of *TaSARD1* transcription and SA biosynthesis [11]. Therefore, it is important to examine the potential interplay among TaSWI3D and CAF-1 in the regulation of *TaSARD1* transcription and SA biosynthesis.

### 4.2. Potentials and Strategies for Exploiting Susceptibility Gene TaSWI3D in Wheat Breeding Against Powdery Mildew Disease

As summarized by previous studies, a plethora of *susceptibility* (*S*) genes have been identified to facilitate wheat compatibility with adapted pathogens [18,19,20]. These *S* genes could regulate various processes in wheat–pathogen interactions, including pathogen penetration, plant defense, and pathogen sustenance [18,19,20]. For instance, wheat S gene *TaECR* is involved in wax biosynthesis, which is essential for stimulating *B.g. tritici* conidial germination, whereas S genes *TaSWP73*, *TaFAS1*, *TaFAS2*, and *TaMSI1* negatively regulate wheat defense response against *B.g. tritici* [11,13,21]. In contrast, wheat *S* genes *TaAMT2;3a* and *TaSTP3/6/13*, respectively, encode ammonium and sugar transporters to facilitate the nutrient uptake and sustenance of the wheat stripe rust pathogen [22,23,24,25,26]. Herein, *TaSWI3D* was identified as a novel *S* gene suppressing SA biosynthesis and contributing to compatible wheat–*B.g. tritici* interaction.

Manipulating *S* genes via genome editing and TILLING techniques could enhance wheat disease resistance [27,28,29,30,31]. For instance, editing of *S* genes *TaWRKY19* and *TaPsIPK1* using CRISPR-Cas9 systems improves wheat resistance against wheat stripe rust disease [32,33]. Similarly, editing of wheat S genes *TaEDR1* and *TaMLO* using CRISPR-Cas9 and transcription activator-like effector nucleases (TALENs) enhances wheat resistance against *B.g. tritici* [34,35,36]. Knockout of the *S* gene *TaSWI3D* via genome editing and TILLING techniques represents a novel approach in wheat powdery mildew resistance breeding. To this end, the potential pleiotropic effects of the *TaSWI3D* gene on other agronomic traits of wheat, which would likely be impacted by knocking out this gene, should be carefully analyzed in future research.

## 5. Conclusions

Herein, we identified wheat *TaSWI3D* as an *S* gene that positively contributes to wheat susceptibility to *B.g. tritici* and revealed that the chromatin remodeler TaSWI3D maintains epigenetic suppression of the SA biosynthesis activator gene *TaSARD1* and negatively regulates SA biosynthesis, thereby positively contributing to wheat powdery mildew susceptibility. This study revealed novel epigenetic regulators governing wheat SA biosynthesis and identified a new *S* gene for wheat resistance breeding against the *B.g. tritici* pathogen. In future research, characterizing the potential interplay of TaSWI3D with other SA biosynthesis regulators like TaSWP73 and CAF-1 could provide more insight into the regulatory mechanisms underlying wheat SA biosynthesis. In this study, we employed transient silencing or overexpressing techniques to characterize the function of *TaSWI3D* in the regulation of compatible wheat–*B.g. tritici* interactions. Generating stable *taswi3d* gene mutants using TILLING or CRISPR-Cas9 techniques not only helps to further reveal the functions of *TaSWI3D* in wheat development and environmental adaptation but could also provide a new direction for wheat powdery mildew resistance breeding.

## Data Availability

The original contributions presented in this study are included in the article. Further inquiries can be directed to the corresponding author.
